# Efficacy of a perfused cadaver model for simulated trauma resuscitation in advanced surgical skills training

**DOI:** 10.1186/s12893-022-01754-1

**Published:** 2022-08-08

**Authors:** Tongporn Wannatoop, Rosarin Ratanalekha, Wanchai Wongkornrat, Kris Keorochana, Parkpoom Piyaman

**Affiliations:** 1 Division of Trauma Surgery, Department of Surgery, Faculty of Medicine Siriraj Hospital, Mahidol University, 2 Wanglang Road, Bangkoknoi, Bangkok, 10700 Thailand; 2 Department of Anatomy, Faculty of Medicine Siriraj Hospital, Mahidol University, Bangkok, Thailand; 3 Division of Cardio-Thoracic Surgery, Department of Surgery, Faculty of Medicine Siriraj Hospital, Mahidol University, Bangkok, Thailand

**Keywords:** Efficacy, Perfused cadaver model, Simulation, Surgical education, Trauma resuscitation, Advanced surgical skills training

## Abstract

**Background:**

To develop a perfused cadaveric model for trauma surgery simulation, and to evaluate its efficacy in trauma resuscitation advanced surgical skills training.

**Methods:**

Fourteen fourth-year general surgery residents attended this workshop at Siriraj Hospital (Bangkok, Thailand). Inflow and outflow cannulae and a cardiopulmonary bypass pump were used to create the perfusion circuit. Inflow was achieved by cannulating the right common carotid artery, and outflow by cannulation of both the right common femoral artery and the internal jugular vein. Arterial line monitoring was used to monitor resuscitation response and to control perfusion pressure. The perfusion solution comprised saline solution mixed 1:1 with glycerol (50%) and water with red food dye added. Advanced surgical skills during life-threatening injuries and damage control resuscitation operations were practiced starting from the airway to the neck, chest, peripheral vessels, abdomen, and pelvis. Resuscitative endovascular balloon occlusion of the aorta (REBOA) was also practiced. Post-workshop survey questions were grouped into three categories, including comparison with previous training methods; the realism of anatomical correlation and procedures; and, satisfaction, safety, and confidence. All questions and tasks were discussed among all members of the development team, and were agreed upon by at least 90% of experts from each participating medical specialty/subspecialty.

**Results:**

The results of the three main groups of post-workshop survey questions are, as follows: (1) How the training compared with previous surgical training methods—mean score: 4.26/5.00, high score: 4.73/5.00; (2) Realism of anatomical correlation and procedures—mean score: 4.03/5.00, high score: 4.60/5.00; and, (3) Satisfaction, safety, and confidence—mean score: 4.24/5.00, high score: 4.47/5.00.

**Conclusion:**

The developed perfused cadaveric model demonstrated potential advantages over previously employed conventional surgical training techniques for teaching vascular surgery at our center as evidenced by the improvement in the satisfaction scores from students attending perfused cadaveric training compared to the scores reported by students who attended earlier training sessions that employed other training techniques. Areas of improvement included ‘a more realistic training experience’ and ‘improved facilitation of decision-making and damage control practice during trauma surgery’.

## Background

The outstanding distinctions that differentiate trauma care from other types of care is that every case is always different, and that the emergency and unstable hemodynamic status of the patient requires fast and appropriate tactical planning to prevent death. In an educational setting that is specific to the teaching of emergency surgical care skills, there are certain limitations that need to be overcome, such as a limited case volume, vast differences among cases, and an assortment of unpredictable variables. The training of essential emergency surgical skills requires a more controlled and less dynamic setting so that foundational knowledge and skills can be developed. Simulation training is currently the best and most widely used method of training in this educational setting [1, 2].

Many simulation training methods have been developed to teach surgical skills, including computerized simulation, video simulation, mannequin-based simulator, and cadaveric model [1–4]. Surgical skills training is broadly stratified into two levels—basic and advanced. When teaching surgical skills of any level, the realism of the teaching method, the setting, and the context is important for instilling skills, overcoming fear, and improving learner confidence and competency to more quickly translate those skills to real-life clinical practice.

Perfused cadaver simulation is being increasingly applied for advanced surgical skills training, especially for procedures that require levels of realism that are similar to real life [4–12]. Importantly, perfused cadaver simulation facilitates student exposure to blood flow, which vastly broadens the scope of both basic and advanced surgical skills learning. Moreover, the realism of blood flow in a perfused cadaver simulation learning setting enhances surgical proficiency by teaching students how to cope with uncontrolled bleeding, how to improve decision-making, and how to manage prioritization of injuries [5, 7–13].

In an attempt to provide improved emergency surgical skills training to surgical residents/fellows at the Faculty of Medicine Siriraj Hospital, Mahidol University—Thailand’s largest national tertiary referral center—the aim of this study was to develop a perfused cadaveric model for trauma surgery simulation, and to evaluate its efficacy in trauma resuscitation advanced surgical skills and damage control surgery training.

## Methods

### Study and post-workshop survey design

This study and the study workshop were conducted at the Faculty of Medicine Siriraj Hospital, Mahidol University, Bangkok, Thailand. The protocol for and the survey used in this study were both approved by the Siriraj Institutional Review Board (SIRB), and the workshop participants all provided written informed consent to participate. A total of 14 4th-year general surgery residents were enrolled.

The survey developed for use in this study was created in Thai language in order to clearly communicate with and elicit information from the workshop participants, all of whom were Thai. The survey was then translated into English language for international journal publication.

The post-workshop survey items and aspects were developed via discussion among the members of the perfused cadaver development team. All team members are experts in their field, and the fields represented on our team included trauma surgery, cardiovascular-thoracic surgery, and anatomy. Moreover, all team members are board certified and have more than 5 years’ experience. All adopted questions, tasks, and skills were discussed among all members of the development team, and were agreed upon by at least 90% of experts from each participating medical specialty/subspecialty. The aim of the developed survey items was to elicit information specific to the following aspects: (1) How the training compared with previously experienced surgical training methods; (2) The realism of anatomical correlation and procedures; and, (3) Student opinion relative to satisfaction with the training, safety, and confidence gained from the training. Each survey item was graded by the workshop participant using a 5-option Likert scale, with a score of 0 reflecting strong disagreement, and a score of 5 reflecting strong agreement. Surveys were distributed to workshop participants at the end of the workshop, the responses were completely anonymous (no name or student number), and the completed surveys were deposited into a closed box. The means of all line items and of the three broad survey categories were calculated. The survey categories and items are listed in Table 2.

### Creation of the perfused cadaveric model

The perfused cadaveric model was developed via a collaborative effort among anatomists, trauma surgeons, and cardiovascular-thoracic surgeons. Fresh human cadavers were prepared by a team of anatomists from the Department of Anatomy of the Faculty of Medicine Siriraj Hospital, Mahidol University. All cadavers were donated, and were tested negative for human immunodeficiency virus (HIV), hepatitis B, hepatitis C, and for syphilis via Venereal Disease Research Laboratory (VDRL) test.

**Fig. 1:**
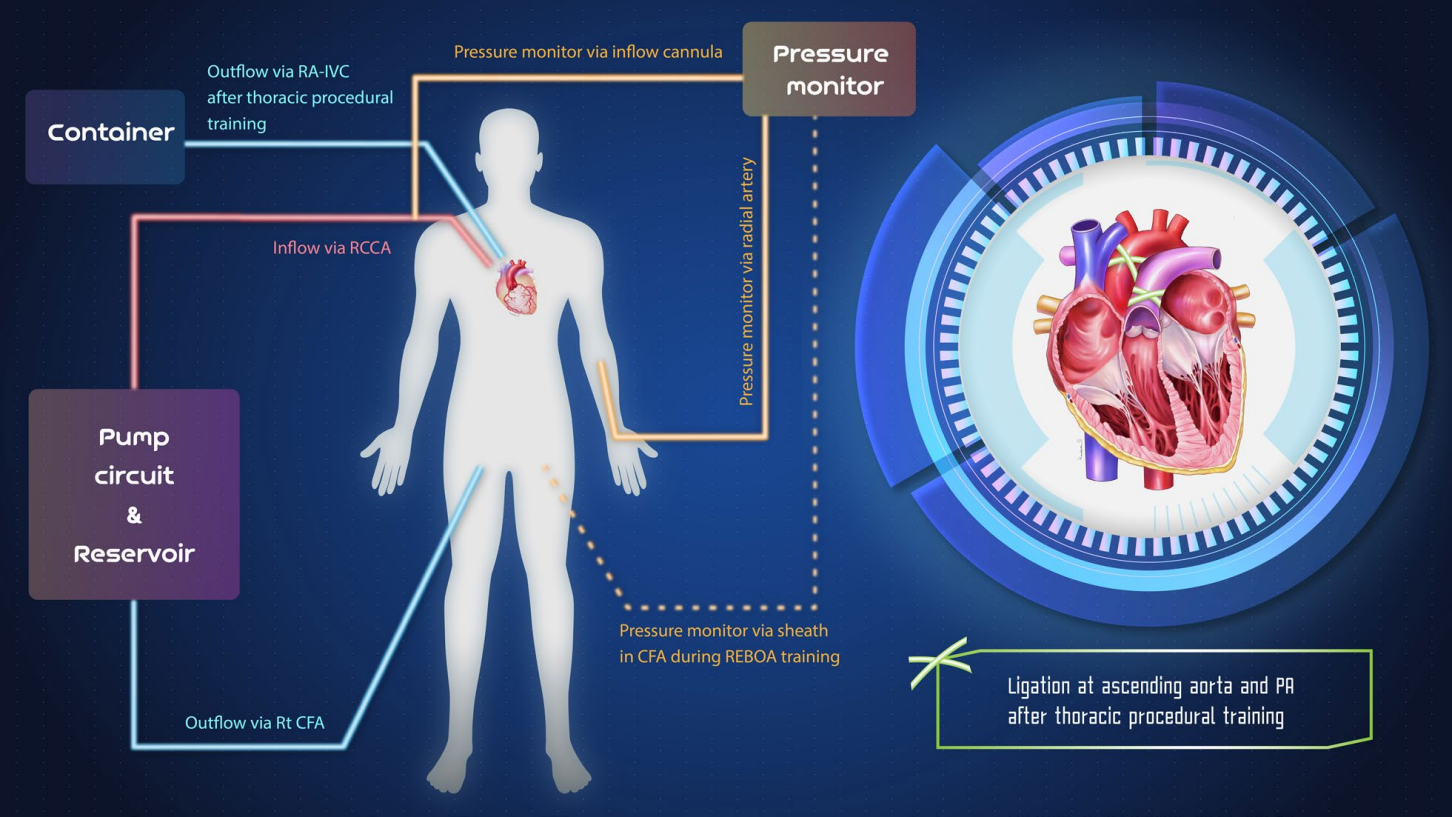
Illustration of the perfused cadaveric model. (*RA* right atrium; *IVC* inferior vena cava; *RCCA* right common carotid artery; *Rt* right; *CFA* common femoral artery; *PA* pulmonary artery; *REBOA* resuscitative endovascular balloon occlusion of the aorta) (Figure provided courtesy of Dr.Tongporn Wannatoop, Bangkok, Thailand)

The perfusion circuit comprised an inflow system and an outflow system. Inflow cannulation was inserted via an open wound into the right common carotid artery. Outflow cannulation was introduced using the same method into the right common femoral artery and the internal jugular vein. The centrifugal pump (Sarns Delphin Cardiac Centrifugal Pump; Terumo Cardiovascular Group, Ann Arbor, MI, USA) was controlled by adjusting the flow to the dynamic procedural requirement or arterial pressure monitoring. Arterial line monitoring was connected to the side connection port of the inflow cannula (Fig. 1). The perfusion fluid was created by mixing glycerol (50%) with tap water with red food dye added at a 1:1 ratio to simulate the appearance of blood. A reservoir was used to hold a sufficient volume of perfusion fluid to compensate for loss of fluid due to leakage and simulated bleeding from study cadavers. The flow rate was adjusted to maintain arterial line pressure within approximately 60–100 mmHg to simulate an exsanguination scenario. For example, to create a profound shock simulation, we set the pressure to very low during resuscitative balloon occlusion of the aorta (REBOA) practice. To practice management of active bleeding, the pressure was set higher. An illustration of the perfused cadaveric model in shown in Fig. 1 just below.

### Development of the workshop trauma procedural agenda

**Fig. 2:**
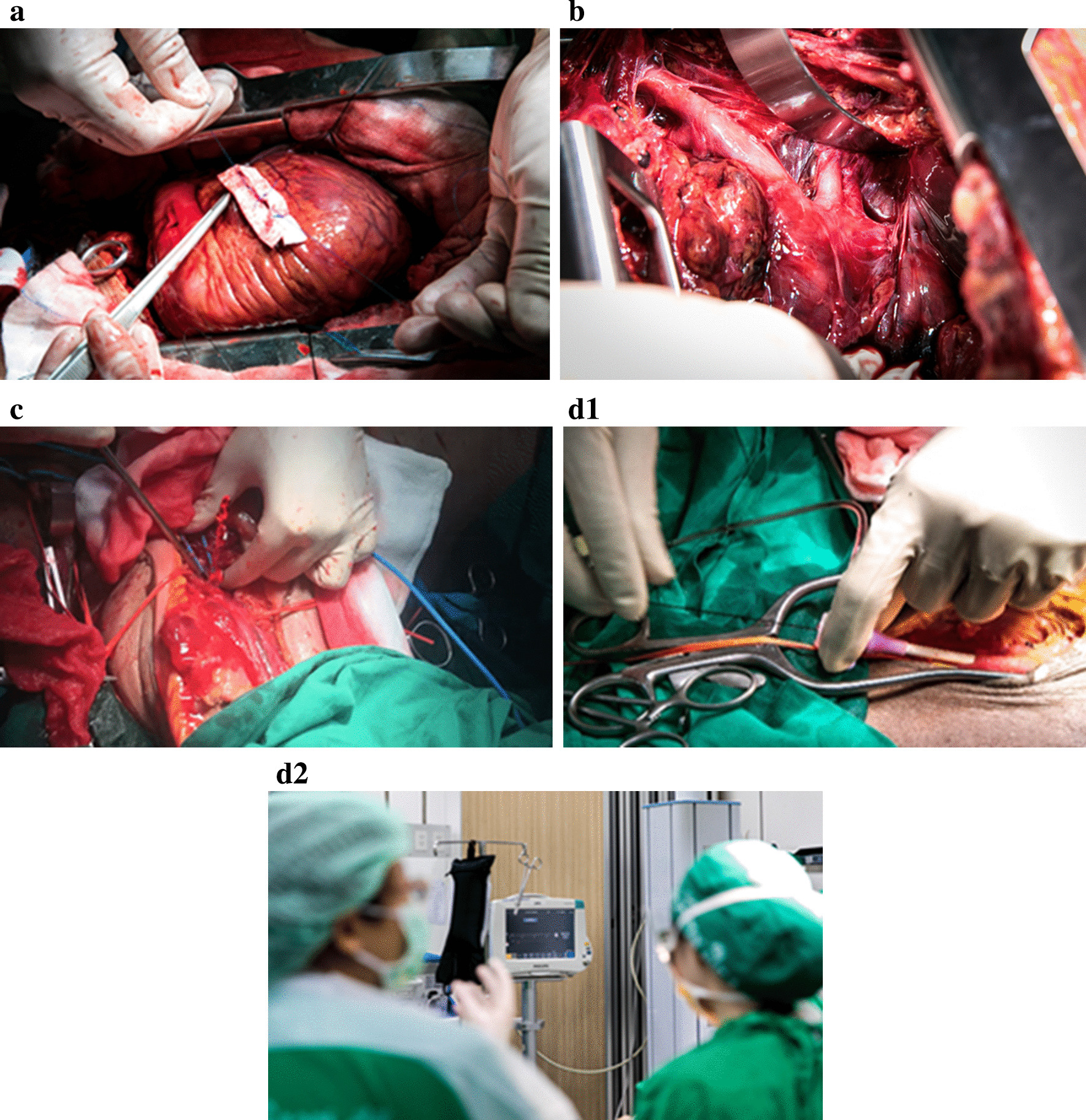
**A** Demonstration of cardiac repair with felt strip reinforcement during manually simulated beating heart. **B** Exposure of arch branches via sternotomy. **C** Simulated vascular injury with pulsatile bleeding at brachial artery. **D1**, **D2** Practicing resuscitative endovascular balloon occlusion of the aorta (REBOA) with pressure monitor

The workshop was designed to include all of the essential resuscitative procedures associated with the advanced surgical skills that are needed to manage life-threatening injuries. Damage control surgery (DCS) starting from the airway and then to the neck, chest, abdomen, pelvis, and peripheral vessels was demonstrated and practiced. REBOA was also taught and practiced (Fig. 2). The order of procedures was designed to minimize the effect of the limitations of the perfused cadaveric model, which is primarily tissue edema of the internal organs, especially the bowel. At the beginning of the workshop, the 14 study participants all sat for a pre-test examination of anatomical knowledge and received a briefing on the surgical techniques to be used during the workshop. These activities were designed to highlight and foretell the areas of study that would be focused upon during the workshop. After the pre-test examination and briefing, the study participants proceeded to hands-on learning and practice of the scheduled procedures while wearing the same personal protective gear that is used by clinicians in a real-world trauma surgery setting. Students were debriefed, questions were answered, and advice was given during the workshop by the two staff surgeons supervising each cadaver station. After the workshop, students were debriefed by all supervisory staff members, a formal question and answer session was conducted, and feedback and advice were provided.

The workshop agenda is shown in Table 1 below. Images of workshop-related activities and procedures can be observed in Fig. 2.

**Table 1:** Siriraj Surgical Trauma Skills (SSTS) Workshop agenda

Scope	Procedure
Life-threatening and damage control surgery (DCS)	Cricothyroidotomy
	Emergency resuscitative thoracotomy
	DCS in chest: pulmonary hilar twist, tractotomy, cardiac injury
	DCS in abdomen: packing, aortic clamping, retrohepatic IVC injury, hilar control for spleen and kidney
	DCS in vessel injury: vascular shunt, fasciotomy
	Demonstrations: resuscitative endovascular balloon of the aorta (REBOA), intraoperative angiography
Thorax	Cardiac box injury: subxiphoid window, median sternotomy, cardiac repair, exposure of great vessels
	Diaphragmatic repair
	Proximal control for aortic injury (optional)
Abdomen	Preperitoneal pelvic packing (PPPP)
	Supra-celiac aortic cross clamp
	Exposure: Lesser sac exploration, retroperitoneal hematoma zone, intra-abdominal aorta
	Solid organ: splenorrhaphy, splenectomy, kidney-nephrectomy, liver-hepatic bleeding control hollow viscus organ: stomach/duodenum/colon repair (optional)
Vascular	Peripheral vascular exposure:
	Neck—carotid vessel
	Upper—subclavian, brachial
	Lower—iliac, femoral, popliteal
	Intra-abdominal aorta

*DCS* damage control surgery; *IVC* inferior vena cava; *REBOA* resuscitative balloon occlusion of the aorta; *PPPP* pre-peritoneal pelvic packing

## Results

Regarding the post-workshop survey, the three categories of questions, the 18 survey items, and the mean results of each are shown in Table 2 below. All 14 student surgeon participants completed and submitted a completed survey, and all mean scores, including those for the three categories and those for the 18 survey items, were above 3.5 out of a possible 5 points. The mean score among the three categories was 4.18, and the mean score among the 18 survey items was 4.30. The highest and lowest mean scores among the three categories were 4.26 (section 1) and 4.03 (section 2), respectively. The highest and lowest mean scores among the 18 survey items were 4.73 (× 2, section 1) and 3.53 (section 2). The mean score among the survey items in section 1, section 2, and section 3 was 4.26, 4.03, and 4.24, respectively. Only 3 of the 18 survey items were rated as lower than 4, and 7 survey items were rated higher than 4.5, with 6 of 7 being category 1 questions. The two most highly rated survey items were ‘Abdominal surgery’ and ‘Vascular surgery’, and both were rated as a 4.73 out of 5. The second most highly rated survey item was ‘Improved ability to develop a mental picture of the case, and a mental strategy for operative treatment’, which was rated at a 4.67. This is important because this suggests the value of this workshop strategy for improving planning and decision-making in a high-pressure scenario. The three lowest survey item scores were given for ‘Circulation mode’ (3.50), ‘Surgical procedure’ (3.93), and ‘Increased surgical skills for hemorrhagic control’ (3.93). This feedback will help us improve certain areas that received lower scores from workshop participants.

**Table 2:** Post-workshop survey items, and the mean score for each item and category out of a possible 5 points

Post-workshop survey	Mean score
**How the training compared with previously experienced surgical training methods**	**4.26**
I gained understanding of surgical techniques of emergency trauma surgery	4.60
I increased my surgical skills in emergency trauma surgery	4.53
I improved my ability to develop a mental picture of the case, and a mental strategy for operative treatment	4.67
I gained surgical skills in each of the following regions:	
Surgical airway	4.47
Thoracic surgery	4.60
Abdominal surgery	4.73
Vascular surgery	4.73
I increased my surgical skills for hemorrhagic control	3.93
I developed decision-making skills for use in an emergency trauma surgery setting	4.13
**Realism of anatomical correlation and procedures**	**4.03**
Realism of the closeness of the feeling of perfused cadaveric tissue to living tissue compared to other learning methods	4.07
The realism of anatomical correlation	4.60
The realism of anatomical dissection during procedure	4.00
The realism of surgical procedures	3.93
The realism of the circulation model	3.53
**Satisfaction, safety, and confidence**	**4.24**
I gained confidence in performing emergency trauma surgery	4.47
I gained safety knowledge in how to safely perform emergency trauma surgery	4.13
What I learned is applicable to real-life clinical practice in the future	4.27
I gained increased belief in myself that I can perform trauma surgery/will become a trauma surgeon	4.07

## Discussion

Advanced simulation is being increasingly applied in medical training to facilitate improved confidence and competency, especially in trauma surgery education [2, 3, 5–7, 10]. Surgical simulation improves psychomotor skills, which when combined with other types of learning develops comprehensive knowledge and skills that can be more quickly and proficiently applied to real-life clinical practice.

This was an experimental study to determine if the perfused cadaveric model developed by our team satisfies the comprehensive agenda for essential surgical skills practice. In this report, we set forth to simply and clearly describe both the model and the workshop agenda so that others who have to work within the constraints of a limited resource setting can adopt this training technique if they desire to do so. The workshop was conducted by a dedicated team that received no additional earnings. Moreover, since this project was unfunded, necessary materials for every aspect of this project were collected from and donated by the three divisions/departments involved in the study.

Although several studies have been conducted in the perfused cadaver model, the benefits of the present study can be listed, as follows: (1) We successfully created an unfunded perfused cadaver model via scavenging of materials from our center and using expertise and labor from our center that received no extra pay; (2) The developed perfused cadaver model satisfies the comprehensive agenda for essential surgical skills practice; (3) We set forth in this report to simply and clearly describe the model and the workshop training techniques so that this model can be easily replicated by other centers or institutions in limited resource settings that may wish to develop a similar model; (4) The model profiled in this report was developed by a multidisciplinary team, including trauma surgeons, cardo-thoracic surgeons, and anatomists; and, (5) When designing this model, we endeavored to create as close to a real-life surgical scenario to enhance surgeon knowledge, decision-making, and confidence.

The perfused cadaveric model simulation method, which was introduced to improve the level realism in anatomical approach and professional skills training, has been implemented in various fields of care, including advanced surgical skills, endovascular procedures, and complex and critical scenarios [4, 5, 7, 8, 10–21]. The achievement of hemostasis in military tourniquet training obtained using the perfused cadaveric model was superior to that obtained from other tourniquet training techniques [22]. The important advantages of this circulation model include pulsatility, realism of vessel dissection, tactile feedback, and simulated bleeding from the injured site at vessel and organ—all of which facilitate the practice of decision-making when surgically treating an unstable patient [10, 11]. The perfused cadaver model continues to evolve, and it has now been developed for out-of-hospital battlefield medical training [15].

We introduced this trauma surgery training method to improve intraoperative decision-making and surgical performance in complex injury cases that require immediate control. Given the very limited amount of available time to manage these cases before the patient expires requires accurate and fast decision-making, including proper prioritization of treatment in cases with multiple injuries, and selection of the right procedure(s). Having or not having these skills is a major determinant when attempting to predict if a surgeon has what it takes to become a trauma surgeon.

The type of cadaver selected depends on the type of procedure to be demonstrated and practiced, and the level of complexity of that procedure. Basic skills include endotracheal intubation, central line insertion, cutdown, and diagnostic procedures. Examples of advanced skills include emergency surgery, complex surgery, surgical exposures in limbs or complex visceral procedures, head and neck flap reconstruction surgery, and brachial plexus surgery. There is still a knowledge gap regarding the best way to demonstrate and practice bowel anastomosis, vascular anastomosis, and laparoscopic procedure training. In the present study, we decided to use fresh cadavers, which have less tissue stiffness for enhanced tissue dissection. These emergency surgical skills are defined as advanced skills, so we decided to focus on as close to a realistic experience as possible, and improvement in both hand–eye coordination, decision-making. To achieve those objectives and even though a direct comparison between models was not conducted, we hypothesized that the perfused cadaveric model would confer more advantages than a simulation machine model.

Planning of techniques and procedures for this workshop had to be carefully considered and developed by taking into account all related factors, especially coordination between the instructor team and the perfusionist team. For example, the cannulation site at the neck vessel was performed on the side opposite the extended cervical incision from sternotomy to the anterior border of the sternocleidomastoid muscles for mediastinal exposure of neck vessels. In addition, the realism of pulsatile exsanguination could be established near the vessel of interest to facilitate practice achieving surgical control and to practice decision-making when the surgeon observed bleeding.

Many cadaver perfusion strategies have been reported [4, 8, 10, 11, 14, 16]. In our study, we initially planned to locate the inflow probe proximal to the simulated vessel sites; however, this was found to be unfeasible due a shortage of time. Even though it demonstrated satisfying pulsatile flow in the upper arm, ascending aorta, and arch branches (all of which were close to the inflow cannulation), we unfortunately encountered a fluid leakage problem that adversely affected our ability to create peripheral vascular pulsatility in the lower extremities. To enhance the realism of vessel flow, a focus on the area of practice is important, such as clamping proximally and distally to the area of interest [18]. We did not apply an arterial balloon to create rhythmic pulsation in a peripheral artery due to the complexity of this procedure. Other techniques that have been introduced to simulate pulsatility include proximal balloon occlusion or manual control by clamping the inflow cannula [10].

Although flow can be generated using water-based solutions, viscous solutions, such as paraffin perliquidum, paraffinum liquidum, and polyethylene glycol, are preferred due to the benefits of their viscosity, osmolarity, and molecular weight. These features were also reported to lengthen intravascular retention time and reduce extravasation [8].

The outcomes of interest in this study were the realism and efficacy of the perfused cadaver model compared to previous training methods used at our center to teach advanced surgical skills. The mean scores of 15 of 18 survey items were above 4 out of 5, and the mean scores of all 3 survey item categories were above 4 out of 5. Interestingly, the abdominal surgery and vascular surgery survey items were scored the highest by workshop study participants (mean score for both: 4.73). Previous studies reported that a focus on organ-specific procedures when using a perfused cadaveric model enhances decision-making in bleeding-related procedures and dynamic scenarios [5, 8, 18]. However, the realism of simulated circulation in our study received the lowest score of 3.53.

A challenge that we encountered during the early part of the workshop was inconsistency among the instructors regarding how to create the perfusion circuit. Our failure to establish a clear protocol prior to the workshop resulted in the creation of some confusion and lost time due to the need to train instructors. This very clearly highlighted the essential importance of training, discussion, and coordination among all members of the workshop training team.

Another challenge was that our model developed tissue leakage with resulting edema. This problem was also previously reported [5, 7, 8, 21]. Measures that have been reported to remedy this tissue leakage and edema outcome include perfusing only as needed during simulated bleeding or vascular procedure, adjusting the flow of fluid with a pressure monitor, ligation of the pulmonary artery to reduce flow to lungs, bilateral iliac vein clamping, selective flow to a specific area of the body where a procedure is being performed, or use of the air perfusion technique [4, 8, 10, 11, 16, 18].

This report describes the first phase of implementation of the perfused cadaver model at our center. Future workshops that use improved versions of our emergency surgical skills training workshop protocol will also benefit from more time spent by workshop facilitators to go over and discuss all aspects of the workshop before the workshop. Facilitators were reported to be essentially important for creating the best learning experiences for students when using the perfused cadaveric model [9, 10].

Regarding assessment of the effectiveness of the perfused cadaveric model, previous studies reported the feasibility, reliability, and validity of this advanced method [4, 8]. Subjective questionnaires using a Likert scale to evaluate realism and the learning experience were used in many studies, including in our project [4, 5, 8, 13]. However, more study of this surgical training model is needed before it can be implemented as part of a surgical training curriculum [4, 10].

We are now at work attempting to improve the components of the workshop that received comparatively lower scores on the post-workshop survey. We also plan to improve the post-workshop survey so we can elicit more information, and more specific information. Lastly, we intend to include more emphasis on decision-making, leadership, and the management of emotions in a high-pressure emergency damage control surgery setting.

### Limitations

This study has some mentionable limitations. First, our study had an experimental/observational design, and there was no direct comparison of our perfused cadaver model with any other commercially available training models. It is, therefore, difficult to report with confidence the efficacy of our model compared to other training models. Second, this model, including how it works, its capabilities, and its limitations, was new and previously unexplored by our team. As such, various minor complexities developed at certain stages of the workshop that were unforeseen. Third, although we used input from our expert assembled team to develop the model, the workshop agenda, and the survey questions, we did not collect expert feedback after the workshop from the experts on our team. Fourth and last, this is a newly internally developed training method that has never before been used at our center. Accordingly, as the training process unfolded, the limitations of our model and our training techniques were revealed. One of the problems that we encountered was a lack of appropriate coordination between the supervisory staff in each group and that group’s perfusion team. Having not done this before would have made predicting such an outcome difficult; however, what we have learned and now know will be integrated into future workshops using our perfused cadaver model.

## Conclusion

The developed perfused cadaveric model demonstrated potential advantages over previously employed conventional surgical training techniques for teaching vascular surgery at our center as evidenced by the improvement in the satisfaction scores from students attending perfused cadaveric training compared to the scores reported by students who attended earlier training sessions that employed other training techniques. Areas of improvement included ‘a more realistic training experience’ and ‘improved facilitation of decision-making and damage control practice during trauma surgery’. Further study is needed to compare the cadaveric perfusion model profiled in this report with another available cadaveric trauma resuscitation model.

## Data Availability

The datasets and analysed generated for the current study are available from the corresponding author on reasonable request due to privacy or other restrictions.

## Abbreviations

CFA: Common femoral artery
DCS: Damage control surgery
ERT: Emergency resuscitative thoracotomy
IVC: Inferior vena cava
PA: Pulmonary artery
PPPP: Pre-peritoneal pelvic packing
RA: Right atrium
RCCA: Right common carotid artery
REBOA: Resuscitative endovascular balloon occlusion of the aorta
Rt: Right
SIRB: Siriraj Institutional Review Board
VDRL: Venereal Disease Research Laboratory
